# Seascape ecology of juvenile gadoid nursery areas

**DOI:** 10.1098/rsos.250705

**Published:** 2025-09-10

**Authors:** Natasha Walker-Milne, Sophie Anne Marie Elliott, Peter J. Wright, David Mark Bailey

**Affiliations:** ^1^ School of Biodiversity, One Health & Veterinary Medicine, University of Glasgow, Glasgow, UK; ^2^ Game and Wildlife Conservation Trust, Fordingbridge, UK; ^3^ Marine Ecology and Conservation Consultancy, Ellon, UK

**Keywords:** stereo baited remote underwater video, Atlantic cod (*Gadus morhua*), habitat modelling, species distribution modelling, haddock (*Melanogrammus aeglefinus*), whiting (*Merlangius merlangus*), heterogeneity, landscape

## Abstract

Availability of juvenile fish habitat provision can impact recruitment. This study focused on identifying which environmental variables characterize the juvenile habitats of three commercially important gadoid species: Atlantic cod (*Gadus morhua*), haddock (*Melanogrammus aeglefinus*) and whiting (*Merlangius merlangus*). Stereo baited remote underwater video surveys were conducted in the South Arran Marine Protected Area between 2013 and 2019 to collect presence/absence data on juvenile gadoids (>20 mm <120 mm) and demersal and epibenthic communities. Data were analysed using binomial generalized additive mixed models. The results revealed spatial segregation among species, each favouring distinct habitats. Predictive modelling suggests a substantial increase in presence probability from 0.25 to 0.75 as the inverse Simpson’s diversity index increases, suggesting that biodiversity appears to be associated with species distribution. Boundary regions between seabed types were associated with variation in species distribution, underlining the importance of seascape heterogeneity. This study underscores the importance of conserving and restoring benthic and epibenthic biodiversity across spatially heterogeneous landscapes. Consequently, reducing benthic pressures could promote early survival for these species, thereby supporting broader ecosystem health and fisheries management goals.

## 1. Background

Understanding the habitat of juvenile fishes is essential for the management and conservation of populations, as juvenile life stages can have a large impact on recruitment success [1–4]. In species with a demersal or benthic settlement, protection of the early settlement stages could assist population recovery [5,6]. With the vital role that suitable habitat plays, removal or degradation of these important post-settlement habitats may have a significant impact on populations [2,7,8]. Here, we define habitat as the combination of variables contributing to the characteristics of an area that influence where a species may occupy (e.g. depth, temperature, biodiversity, seabed type, etc.). In this study, seabed type refers to the composition of the substratum [9]. The definition of ‘nursery’ habitats has evolved in recent years, from being described as areas where higher densities of juveniles are found [10], to those that make a higher-than-average contribution to recruitment into the population [11]. Research has also been conducted to understand why some areas are more productive, considering aspects such as the complexities of patch connectivity, ontogenetic shifts and resource availability [12,13]. Habitats, which provide post-settlement refuge and foraging potential, can have a marked impact on the survivability of juvenile demersal and benthic fishes [14]. Understanding how these habitat attributes influence the early life stages of key demersal fishes, such as gadoids, is therefore important for effective management and conservation.

Juvenile fishes often do not rely on a single habitat [8,15], but rather a succession of differing ones, as their requirements change in response to prey availability and predation risk and through not only ontogenetic stages but also daily requirements [16]. Therefore, it is important to consider how a mosaic of habitat types and their arrangement influences post-settlement selection and survival. The ‘seascape nursery’ approach is valuable because it provides a framework for considering both spatial and temporal variation in habitat use, as well as the distinct requirements associated with different ontogenetic stages [13]. Given that survival through these early stages influences population growth [16,17], it is essential that the spatial and temporal scales reflect the ecological needs of the focal species, as requirements vary across species and their life-history phases.

For many species, nursery habitats are typically found in shallow coastal waters, where greater food availability, reduced physical stress and increased shelter enhance juvenile survival [18–21]. The quality of habitat, defined as the extent to which an area provides the species-specific physical, chemical and biological conditions necessary for optimal growth, survival and future reproductive success [9,22], is also important, and often indicated by higher biodiversity. Bottom-contacting mobile fishing gear can have long-term impacts on ecosystem health, including alteration of community structure [23], reductions in benthic biomass [24] and extended post-impact recovery times following removal [25]. This suggests that year-round protection of juvenile fish habitat rather than seasonal closures may be needed to promote recruitment while restoring and maintaining demersal and epibenthic biodiversity.

Epibenthos contributes to habitat complexity and provides food sources to lower trophic levels, subsequently supporting piscivorous predators. This interconnectivity across all trophic levels promotes ecosystem stability and faster recovery from disturbance [26–28]. Areas with high biodiversity are often areas of increased primary and secondary production and increased food sources, leading to higher growth, fecundity and resilience to invasive species [27,29,30]. A number of temperate coastal marine habitats have been associated with areas of increased biodiversity owing to their complex three-dimensional structures and increased foraging opportunities, including seagrass beds [31–33], maerl beds (*Phymatolithon calcareum*) [34–36] and kelp forests [37,38]. Predictor variables for biodiversity vary between biota; thus, each must be considered on a suitable landscape scale for each habitat [39]. Despite the many benefits of increased biodiversity, marine protected areas (MPAs) are often not designed with measurable improvements of metrics in biodiversity as a goal [40,41], and often focus on a specific species or habitat [42,43], despite biodiversity being a specific goal of the United Nations Convention on Biological Diversity [44]. To address this issue, some proposals have been put forward to pivot towards more ‘whole-site’ MPA management [41,45,46], with evidence that network-scale bioregional approaches are more effective for sustaining biodiversity than feature-specific designations [40]. If the focus remains on a selection of designated features such as those included in the Priority Marine Features approach used in Scotland (e.g. maerl beds, flame shell beds) [47], the types of seascapes protected may not be optimal for biodiversity protection or to support juvenile fishes.

Commercially important demersal species, such as Atlantic cod (*Gadus morhua*), haddock (*Melanogrammus aeglefinus*) and whiting (*Merlangius merlangus*), have undergone population declines in several areas of the northeast Atlantic over recent decades, accompanied by changes in community structure [48,49]. Despite efforts to reduce fishing or enact spatial and temporal closures, long-term effects on population size and composition are evident [50–52]. These stocks are currently not achieving their productive potential, and rebuilding them requires a broad range of measures, including well-designed spatial measures [53]. Previous studies have demonstrated tendencies for each species to favour certain juvenile gadoid conditions, such as differences in seabed composition [16,54,55] and depth [56]. All three species display some degree of site fidelity on settlement [14,57–59] and therefore, the availability of undisturbed and suitable nursery areas post-settlement may be a necessary component of successful recruitment and recovery. All three species display similar dietary niches when settlement first occurs and are in the transitional period from pelagic to demersal foraging [58]. As their size increases, there is divergence in dietary needs, although mid-sized *Mel. aeglefinus* and *Mer. merlangus* may still experience interspecific competition [58]. However, detailed dietary analyses indicate that resource partitioning among species and size classes reduces the likelihood of food limitation in nursery areas [60], although changing environmental conditions (e.g. climate-driven shifts in productivity) may increase the potential for competition in some systems. The use of shallower waters may not be a direct avoidance of predation, but rather a strategy to avoid dedicating as much time to vigilance, so more effort can be put into foraging [20].

The Firth of Clyde on the west coast of Scotland has a long history of commercial fishing; there is evidence that fisheries impact in the Clyde has been an ongoing concern for some time. The rise of industrial fishing in the nineteenth century was associated with a decline in most fish stocks [61]; the impacts of which were raised as concerns regarding bottom trawling on benthic spawners and the seabed as early as the 1880s. This heavy exploitation of the Firth of Clyde has led to changes in the community structure of populations, affecting taxonomic richness, evenness and maximum length [49]. Changes in Clyde fishery biomass showed that in the period 1927–1959, 13 taxa were responsible for 95% of biomass, which dropped to four taxa between 1995 and 2004, with *Mer. merlangus* alone accounting for greater than 80% of biomass [49].

In this study, we investigated how habitat variables (including proportional coverage of seabed types, depth, patch characteristics, distance to shore, current velocity) influence the presence of three commercially important juvenile gadoid species: *G. morhua*, *Mel. aeglefinus* and *Mer. merlangus*, at both patch and landscape scales, using stereo baited remote underwater video (SBRUV) surveys [62]. The aim of this study was to enhance the current understanding of juvenile habitat associations for all three species and to generate accurate predictions of species distribution, considering the effects of biodiversity and landscape composition. We focused on the Firth of Clyde in Scotland as a case study and hypothesized that juvenile individuals of each species would exhibit distinct habitat preferences influenced by factors such as foraging behaviour, dietary requirements and predator avoidance strategies.

## 2. Methods

Surveying was conducted in 2013, 2014, 2018 and 2019 around the Isle of Arran, the largest island located in the Firth of Clyde on the west coast of Scotland (55.5806° N, 5.2109° W). Surveying effort and spatial coverage varied between years owing to logistical constraints (equipment availability, vessel access and weather conditions). The dataset was designed to build cumulative spatial coverage rather than to support direct interannual comparison; therefore, year was not included as a variable in analyses. Stretching around Arran’s southern coast is the South Arran Nature Conservation MPA. The South Arran MPA is a multi-use MPA with a series of fishing restriction measures (ranging from prohibition of dredging up to a no take zone) targeted at the protection of certain features, such as maerl beds and seagrass, while also allowing continued use of the area by both commercial and recreational human activities [62,63].

### 2.1. Survey methods and equipment

SBRUV surveys were carried out within the South Arran MPA using a 10.8 m research vessel (RV *Actinia*), 6.5 m rigid inflatable boat, and 10.0 m creel boat. Three SBRUV camera set-ups each consisted of a pair of Canon HF G25 video cameras encased in waterproof camera housings mounted on a steel frame 57 cm in height with an inward angle of approximately 8^o^ and a basal separation of 58 cm [16,64], with two LED W38VR Archon lights providing illumination as per [16]. Frames were freshly baited with locally caught mackerel (*Scomber scombrus*) for each station. The camera systems remained on the seabed and recorded for 60 min per deployment with species recording commencing after a 3 min settlement time. Depths for SBRUV deployments ranged from 4.1 to 47.2 m and were conducted between 07.45 and 17.28 to avoid crepuscular behaviour. To maintain consistency in spatial coverage, SBRUV deployments deeper than 40 m were excluded from the analysis, as only a small proportion of deployments exceeded this depth. The MPA was divided into five zones based on levels of fetch exposure, bathymetry and coastal characteristics [16,62]. Stations were selected within each zone using random points stratified by depth using a quantum geographic information system (QGIS) [65]. SBRUV camera equipment was used in conjunction with drop-down video carried out by NatureScot and the Scottish Environmental Protection Agency to examine and map seabed composition [62].

Videos were analysed using EventMeasure™ software [64] with presence recorded for each fish species and a still image captured from each station for seabed analysis [62]. As SBRUV equipment was used in this survey, it was possible to take stereo measurements of fishes when visible in both cameras. Stereo video calibration and measurement protocols followed Elliott *et al.* [16]; length measurements with a root mean square error of greater than 2 cm or a precision greater than 0.5 cm were excluded. Owing to water clarity and identification requirements, most fishes measured were within 1–2 m of the camera system. This allowed for the confirmation of the 0-group gadoid status with sizes remaining less than 120 mm [35]; *G. morhua* larger than this were observed sporadically but were not recorded for this study as their size indicated 1-group (>200 mm) [66].

**Figure 1.**
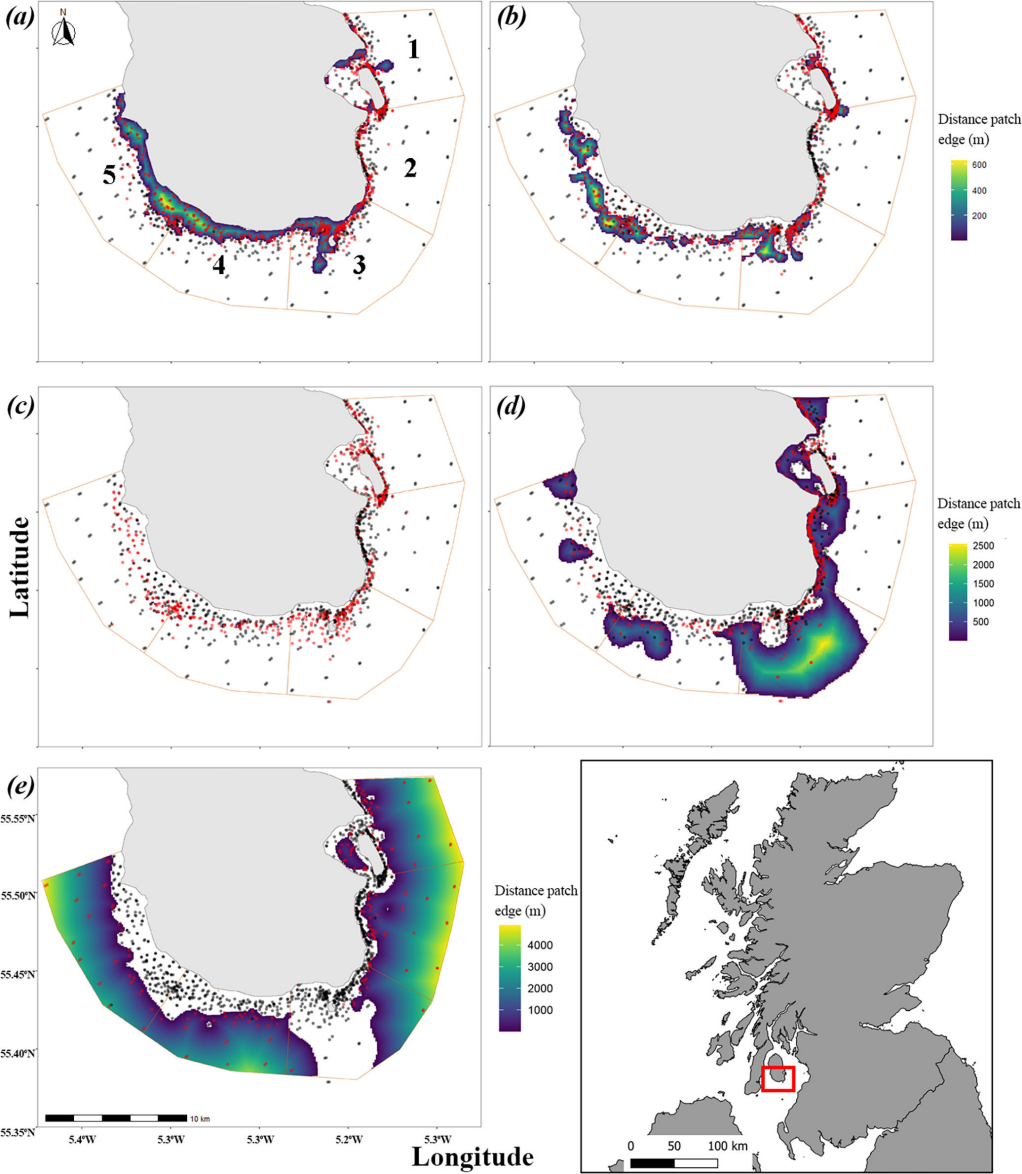
Euclidean distance to the edge of seabed type patches as created from seabed patches produced in [62]. (*a*) Algae, (*b*) gravel, (*c*) pebble, (*d*) sand and (*e*) mud. Positive values denote within-habitat distances with increased values showing distance further into patches; mud is known to extend beyond the boundaries of the MPA [68]. Sampling locations are coloured by seabed type; red points indicate presence and black points indicate absence. Orange lines denote boundaries between MPA zones (numbered 1–5) used in generalized additive mixed models as a random effect.

Environmental variables included proportional coverage of seabed type, classified as mud, sand, gravel (including both alive and dead maerl), pebble and algae (encompassing all types of macroalgae which were attached to the seabed), and seabed patch characteristics such as area (m^2^), perimeter (m) and perimeter-to-area ratio. Euclidean distance to the edge of the patch (m) was also calculated using R statistical software (R v. 4.4.1) [62,67], and post-calculated analysis was conducted in QGIS. This resulted in a positive distance within the patch to the edge of the patch boundary and a negative value for distances outside the patches to allow for a metric of distance to the seabed type patch edges (figure 1). The other model variables were mean current velocity (ms^−1^), distance to shore (km), depth (m) and terrain ruggedness index (TRI; table 1; electronic supplementary material, figure S2). Simpson’s inverse diversity index was calculated for each site using species counts of epibenthic and demersal fishes, and macroinvertebrate species [62], hereafter referred to as biodiversity. Seagrass was excluded from the final models because of the small sample size. As noted previously, the year was not included as a variable in the analyses.

**Table 1.** Summary of environmental model variables, details of seabed classification [62].

variable	unit	description
mud	proportional coverage (0–1)	visual coverage of seabed type in each site from NatureScot and University of Glasgow surveys^a^
algae		
sand		
gravel		
pebble		
distance to shore	km	Euclidean distance to shore for each survey site
depth	m	bathymetry data 1 s arc
terrain ruggedness index (TRI)	in metres of elevation difference for grid points 1 arc-seconds apart	elevation differences between grid points calculated using the raster package in R
inverse Simpson’s diversity index	1/*D* = *N*(*N*−1)/∑(*n*/N)	biodiversity indices^b^
mean current velocity	ms^−1^	mean current data [70]
distance to edge of seabed patch	m	Euclidean distance to patch edge for each survey site
patch perimeter	m	distance around patch area

^a^ Gravel contains maerl, both dead and alive as live maerl was in too small quantities to be classified as separate seabed type.

^b^ Predicted demersal and epibenthic biodiversity calculated in R using rDiversity package [69].

### 2.2. Statistical analysis

Collinearity checks were performed on all variables using multicollinearity diagnostic measures and Spearman’s rank correlation coefficient [71]. Model selection was carried out using backward stepwise selection using Akaike information criterion model evaluation, and smoothing terms for the models were determined through model selection (electronic supplementary material, table S1). The model of best fit was tested against the null hypothesis using a log-likelihood ratio test. The months of sampling were combined into early (June/July, *n* = 201) and late (August/September, *n* = 322). This was included to investigate any interactions with depth and seabed type coverage to examine if there were significant ontogenetic shifts. The model results were checked for spatial autocorrelation using Moran’s I statistic. There was initial evidence of spatial autocorrelation; therefore, the study area was partitioned into zones as described by Elliott *et al.* [16], which then accounted for spatial autocorrelation, and the MPA zone was included as a random effect.

The area under the receiver operating curve for fish species models was calculated for the model of best fit, as was the model’s per cent correctly classified and a confusion matrix was calculated using the *PresenceAbsence* package [72]. Model accuracy testing was conducted on a 25% split of the data using the *caret* R package [73]. Predictions were mapped using a 100 m point grid of environmental variables (electronic supplementary material, figures S1 and S2) [62]. Error maps were produced by calculating the model fit for each grid point and subtracting the binomial presence/absence of the species. The predicted error of species presence/absence was mapped using ordinary kriging with *automap* [74], and the degree of extrapolation for model predictions was calculated using the multivariate environmental similarity surface index (electronic supplementary material, figure S3) using the R *dismo* package [75]. All figures were produced using the *ggplot2* package [76].

## 3. Results

The surveys resulted in 544 SBRUV deployments across all years, of which 33 data points were excluded during initial review after deployment owing to technical issues (such as tipped frames or poor visibility owing to lighting failure), and a further 23 were removed during data cleaning (incomplete metadata, duplicate records), resulting in 488 data points. *Gadus morhua* was most likely to be present when the proportion of seabed covered by algae was approximately 0.5 (*χ²* = 6.49, *p* < 0.0001; table 2). There was also a positive increase in *G. morhua* presence probability with increased gravel coverage, increased sand coverage, TRI and biodiversity.

**Table 2.** Summary of model variables and outputs for 0-group gadoid presence including area under curve (AUC), per cent correctly classified (PCC). (Explanatory variables show proportional coverage of seabed type, depth (m), mean current velocity (ms^−1^), inverse Simpson’s diversity index, terrain ruggedness index (TRI) and distance from survey points to edge of seabed patch edge for sand, mud, algae and gravel (m), distance to shore (m), with MPA zone as a random effect. Coefficients and diagnostics (*X*^2^ and *p*-values) indicate the effect of each parameter, with variables of statistical significance in italics, * = *p* < 0.05, ** = *p* < 0.01, *** = *p* < 0.0001.)

model: *G. morhua*	AUC: 0.85	PCC: 0.77	PCC s.d.: ± 0.02
	zero: 53%	deviance: 30.19	*R^2^:* 0.34
	variable	*χ²*	*P*
	*algae*	*63.49*	<0.0001 ***
	*gravel*	*21.06*	<0.0001 ***
	*sand*	*8.14*	0.004 **
	pebble	2.86	0.091
	*TRI*	*15.42*	<0.0001 ***
	*Simpsons*	*18.38*	<0.0001 ***
	mean velocity	2.22	0.315
	*distance to sand edge*	*11.35*	0.024 *
	*distance to mud edge*	*5.99*	0.014 *

model: *Mel. aeglefinus*	AUC: 0.89	PCC: 0.81	PCC s.d.: ± 0.02
	zero: 56%	deviance: 38.91	*R^2^*: 0.43
	variable	*χ²*	*P*
	*algae*	*39.07*	<0.0001 ***
	*depth*	*68.91*	<0.0001 ***
	*mean velocity*	*4.69*	0.030 *
	*distance to shore*	*6.25*	0.012 *
	*Simpsons*	*13.83*	0.0002 ***

model: *Mer. merlangus*	AUC: 0.89	PCC: 0.81	PCC s.d.: ± 0.02
	zero: 57%	deviance: 39.65	*R^2^:* 0.44
	variable	*χ²*	*P*
	*depth*	*44.25*	<0.0001 ***
	*TRI*	*8.68*	0.003 **
	mud	3.37	0.066
	*mean velocity*	*7.61*	0.005 **
	*Simpsons*	*24.87*	<0.0001 ***
	*distance to algae edge*	*21.48*	0.0002 **
	*distance to gravel edge*	*7.92*	0.005 **

**Figure 2.**
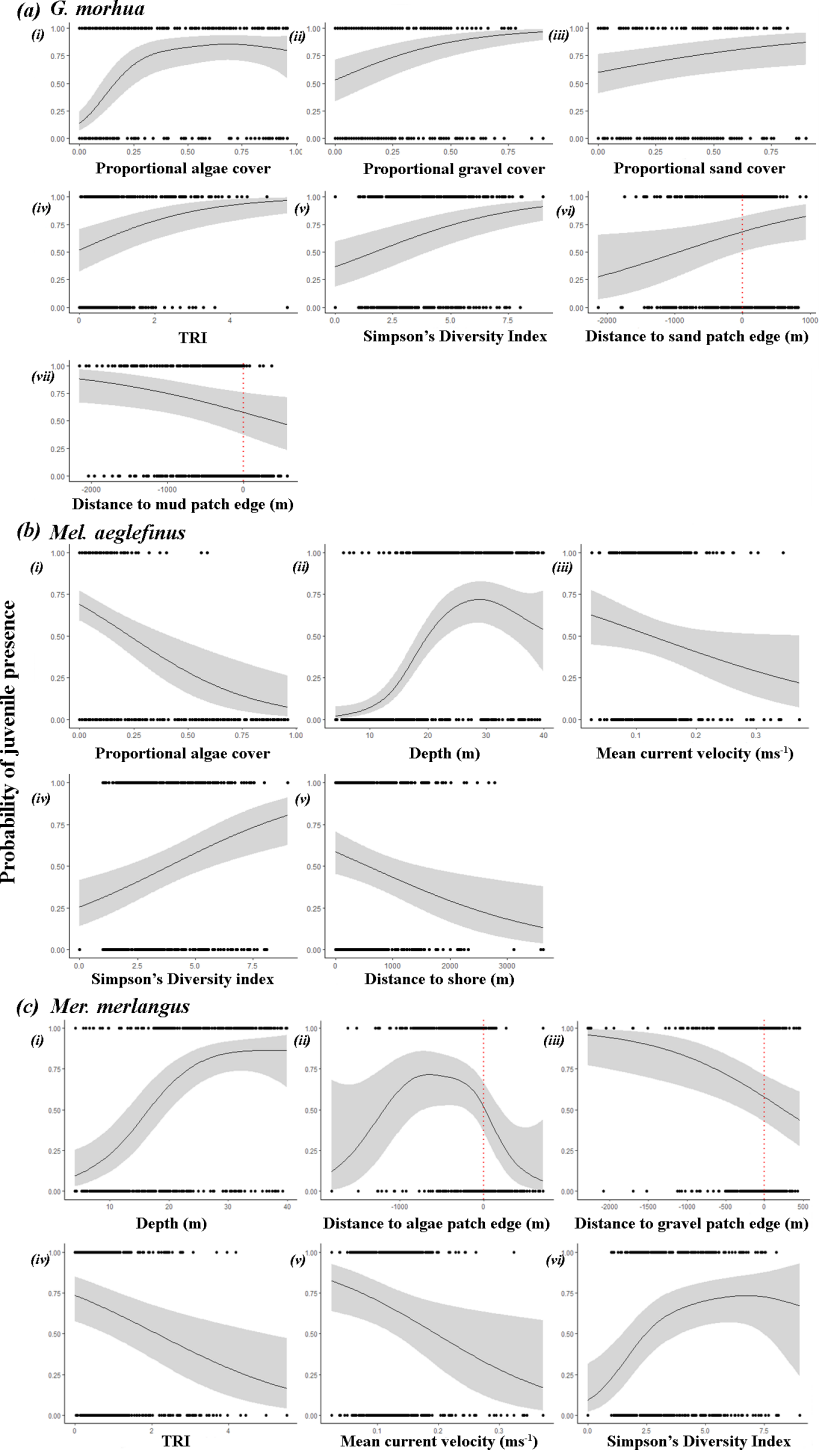
A combined generalized additive mixed model predicted the response of the influences of fixed biotic and abiotic variables on the probability of presence in 0-group fishes: (*a*) *G. morhua – (i) proportional algae cover, (ii) proportional gravel cover, (iii) proportional sand cover, (iv) terrain ruggedness index (TRI), (v) Simpson’s diversity index, (vi) distance to sand patch edge, (vii) distance to mud patch edge.* (*b*) *Mel. aeglefinus* – (i) proportional algae cover, (ii) depth, (iii) mean current velocity, (iv) Simpson’s diversity index, (v) distance to shore. (*c*) *Mer. merlangus* – (i) depth, (ii) distance to algae patch edge, (iii) distance to gravel patch edge, (iv) TRI, (v) mean current velocity, (vi) Simpson’s diversity index Distance values within and outside of patches, positive values are within patches, negative values are outside of patches (m). Grey shaded areas represent 95% confidence intervals, red dashed lines indicate the boundary of within and outside seabed patches.

*Gadus morhua* was also affected by the distance to the edge of the seabed type patch, with sand showing a positive relationship and mud showing a negative relationship (figure 2*a*(*vi*)(*vii*); table 2). Depth was not significant for *G. morhua,* unlike *Mel. aeglefinus* and *Mer. merlangus,* which were both affected by depth. *Melanogrammus aeglefinus* presence peaked between 20 and 30 m depth, whereas *Mer. merlangus* presence was influenced by depth, peaking at approximately 30 m (figure 2*b*(*ii*),*c*(*i*)). There was a negative relationship between *Mel. aeglefinus* presence and increases in algal coverage, mean current velocity and distance to shore (figure 2*b*(*i*)(*iii*)(*iv*)). *Merlangius merlangus* probability showed a negative relationship between the presence and increases in TRI and mean current velocity (figure 2*c*(*iv*)(*v*), respectively). The distance from algal patch edges influenced *Mer. merlangus*, resulting in decreases within algal patches and far outside of algal patch edges, but a peak in probability just outside algal patches (figure 2*c*(*ii*)). All three species showed a positive relationship with increased biodiversity, which was the only shared variable across the three species.

**Figure 3.**
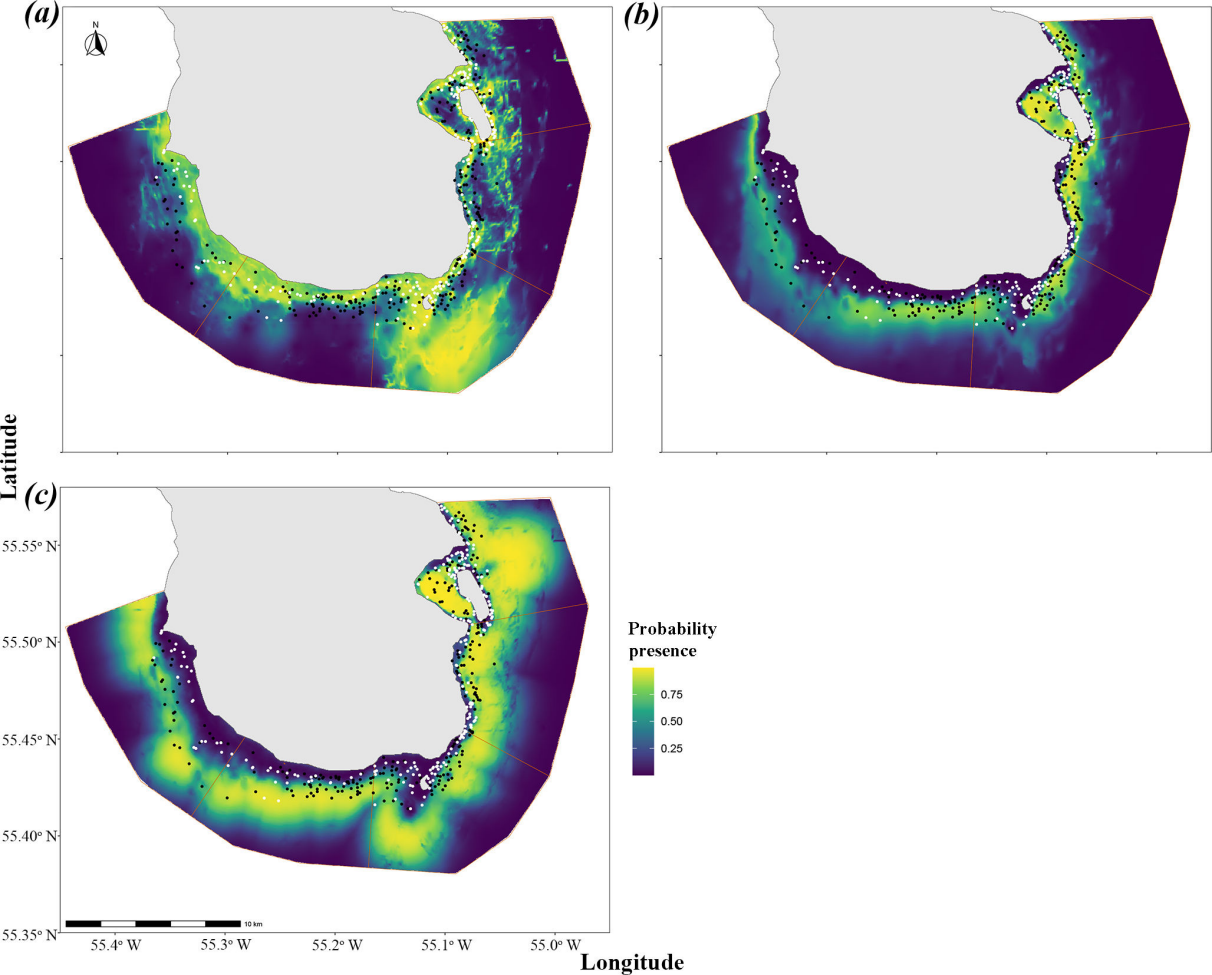
Mapped presence predictions for 0-group gadoids: (*a*) *G. morhua*, (*b*) *Mel. aeglefinus* and (c) *Mer. merlangus* in the South Arran MPA. Species presence as recorded on stereo baited remote underwater video (SBRUV) surveys, black points absent and white points present.

*Gadus morhua* were found in areas associated with high algae coverage to the southwest of the island and in areas of high sand coverage to the southeast (figure 3). *Melanogrammus aeglefinus* were found further away from the coast around the southwest of the island, avoiding areas of high algae coverage, but showed an increased presence in areas on the east coast of the island in deeper waters, both of which have a lower mean current velocity and are close to shore, providing increased shelter. *Merlangius merlangus* were predicted in a band around the island at increased depth away from areas of algal coverage but close to the borders of algal patches. This peaked in distance to the algae patch edge at approximately 500 m outside the patch areas (figures 2 and 3). Although *Mer. merlangus* showed a preference for increased depth and areas with less rugosity, the distance out into deeper water appeared to be limited by the distance from algal patches.

## 4. Discussion

This study demonstrated that the probability of the presence of all three gadoid species was positively associated with increased demersal and epibenthic biodiversity. Although each species responded differently to other environmental variables, biodiversity was the only variable consistently associated with presence across all three species. These results for a temperate marine ecosystem build upon previous investigations into the impact of marine biodiversity on fish biomass and community regulation [77,78]. Identifying cross-species associations is important for informing multispecies management strategies, while recognizing that species-specific habitat requirements will vary between species. Areas with increased biodiversity have been demonstrated to have higher ecosystem services and greater community resilience to pressures and stochastic events [79,80]. This may be facilitated through factors including, but not limited to, genetic diversity and increased species interactions such as complementarity and facilitation [81,82].

The relationship between each species and seabed patch is interesting, as it is not only the degree of seabed coverage that plays a role but also the distance to or from patch edges, with *G. morhua* showing increased presence within sand patches and *Mer. merlangus* showing decreased presence inside gravel patches, as well as peaks in presence around the edges of algal patches (figure 2*a*(*vi*),*c*(*ii*)(*iii*)). Differences in the presence between species can be related to dietary requirements, predator avoidance strategies and size-specific segregation [83–86]. In the absence of predation pressure, juvenile *G. morhua* will select sand or gravel areas and will often forage more efficiently over sand, although this will leave them open to increased predation [55]. Despite this, juvenile *G. morhua* often settle in areas of higher complexity to increase survival [48,87,88]. As juvenile *G. morhua* rely on a predator avoidance strategy through camouflage rather than shoaling, it would be prudent to remain close or within areas of refuge [21]. The need to balance areas of concealment with areas of foraging may explain the preference for boundary areas of sand and areas where sporadic clumps of algae are present within sand patches. The presence of *G. morhua* in areas with a high degree of algal coverage far within a designated sand patch may also be owing to the usage of small clumps of unfastened algae that were not part of the main extent of an algae patch (figure 2*a*(*i*)(*iii*)). Sporadic algal patches have previously been observed to provide intermediary refuges for *G. morhua* transiting an area or indeed gaining access to foraging in open sand, but with a means of concealment, as such gap crossing is mediated by the provision of cover.

Predator avoidance is displayed in juvenile *G. morhua* activity compared with other more generalist species such as *Mer. merlangus*. This may be a result of boldness and more prominent scavenging behaviour exhibited by juvenile *Mer. merlangus,* resulting in a competitive advantage [86]. Such boldness may contribute to sampling bias associated with SBRUV equipment, as typically shy species will leave the field of view when larger or shoaling species arrive [86,89], and when encountering species with a high site fidelity, which may inflate perceived abundance in a small survey area [90]. Although all surveys were conducted within daylight hours owing to practical constraints (e.g. safety and tides), lights were included in the SBRUV set-up to add in species identification. Despite this limitation, SBRUVs have consistently demonstrated relatability in sampling carnivorous and scavenging species without negatively impacting the detection of herbivorous or omnivorous species [91,92]. However, considering the potential for diurnal bias, it is recommended that future studies also conduct nocturnal surveys for a more comprehensive understanding of species assemblages.

*Merlangius merlangus* were more likely to be present over areas with a lower TRI and were less likely to be observed closer to the edges of the gravel patch. The likelihood of the presence of *Mer. merlangus* further declined with increasing distance inside gravel patches. However, the peak in presence around algal patches (figure 2*c*(*ii*)(*iii*)) provides a noteworthy result. Although algal coverage itself does not provide the habitat necessary for juvenile *Mer. merlangus*, areas bordering it provide foraging opportunities that *Mer. merlangus* may find favourable [93]. Predators of juvenile *G. morhua*, such as older gadoids, including conspecifics, have been observed foraging on 0-group *G. morhua* along seagrass patch edges [94], and the same interaction may be present around areas of algae. Interestingly, given the results observed for juvenile *G. morhua*, if they are using algae for predator avoidance and foraging in areas surrounding algae, this could account for the presence of *Mer. merlangus* on this band bordering algal patches. *Merlangius merlangus* have been observed to predate on juvenile *G. morhua* [95]. The spike in presence outside of algal patches has been previously observed in other species, where patch edges attract predators [94]. *Merlangius merlangus* display numerous different habitat preferences [16,93,96]; as such, *Mer. merlangus* may display more plasticity in their preferred juvenile habitat depending on local conditions.

Because algae are only found in shallow water, this may limit the presence of juvenile *G. morhua* in some cases to shallower waters. While *Mer. merlangus* populations exhibit resilience and competitive advantages attributed to their extended settlement period and dominant behaviour, species such as *G. morhua* and *Mel. aeglefinus* display distinct depth preferences, as evidenced by presence patterns observed during this study. Specifically, *Mel. aeglefinus* presence was highly influenced by depth, peaking at approximately 30 m. This is shallower than previous observations in the North Sea, where juvenile *Mel. aeglefinus* were found at much greater depths [56], and could be specific to some deeper inshore areas around Arran. *Melanogrammus aeglefinus* presence was also more likely in areas with low current velocity and close to shore. Juvenile *Mel. aeglefinus* in this study were predicted to be present mainly in areas with lower algal coverage, deeper than 20 m, with lower current velocity, higher biodiversity and close to shore. Although *Mel. aeglefinus* showed a relationship with the proportion of seabed type cover, characteristics such as distance to the seabed patch edge appeared to have no effect. Given the role that specific habitat characteristics, such as patch edges and areas of increased biodiversity, have on juvenile gadoids, ongoing anthropogenic impacts, such as those caused by bottom-contacting mobile fishing, pose a threat to these ecological components. Such activities are known to change community structure and damage the habitat complexity and biodiversity shown in this study to be important to juvenile fish presence.

While SBRUV deployments in this study were primarily limited to the nearshore and shallower areas (<40 m), this was based on both logistical constraints and the known ecology of juvenile gadoids, particularly *G. morhua* and *Mer. merlangus,* which predominantly occupy nearshore and shallow areas during their early demersal life stages [57]. While *Mel. aeglefinus* may show greater use of deeper water, SBRUV deployments in these areas were limited by logistical and safety constraints. The lack of offshore data may under-represent some aspects of spatial distribution, particularly for later stage *Mel. aeglefinus* and *Mer. merlangus*, and we recommend that future studies integrate additional sampling to address this. Nonetheless, our sampling strategy is consistent with both previous studies [16] and ecological understanding of juvenile habitat use [54,57,58] and is unlikely to have introduced substantial bias regarding the post-settlement habitats for the study species. Multivariate environmental similarity surface maps (electronic supplementary material, figure S3) indicate that statistical predictions are robust in the majority of nearshore and environmentally representative areas and decrease in predictive power in offshore regions.

## 5. Conclusion

It is the quality of habitat, through higher biodiversity, which is most strongly associated with the presence of all three species in conjunction with the benefits of boundary effects, which emphasize the importance of heterogeneous habitat mosaics. Our results emphasize the need for a seascape approach to understand habitat usage. It also highlights the importance of the conservation of habitats for commercial species, which would be best achieved through whole-site restrictions on harmful activities. Marine landscape composition and connectivity for mobile species, such as gadoids, would be better served by such whole-site management rather than protection of single features without consideration of their context [45,97–99].

## Data Availability

The data and code that support the findings of this study are available from Dryad [100]. Supplementary material is available online [101].
